# Phase separation of the *C*. *elegans* Polycomb protein SOP-2 is modulated by RNA and sumoylation

**DOI:** 10.1007/s13238-019-00680-y

**Published:** 2020-01-01

**Authors:** Wenyan Qu, Zheng Wang, Hong Zhang

**Affiliations:** 1 School of Life Sciences, University of Science and Technology of China, Hefei, 230027 China; 2 National Laboratory of Biomacromolecules, CAS Center for Excellence in Biomacromolecules, Institute of Biophysics, Chinese Academy of Sciences, Beijing, 100101 China; 3 College of Life Sciences, University of Chinese Academy of Sciences, Beijing, 100049 China

**Dear Editor**,

Macrobiomolecules such as proteins, RNA and DNA can coalesce into liquid-like condensates via liquid-liquid phase separation (LLPS) (Shin and Brangwynne, 2017; Banani et al., 2017; Wang and Zhang, 2019). Phase-separated condensates, also called bodies, granules or membrane-less compartments, have many functions, including temporal and spatial control of signaling and biochemical reactions, storage of essential cytoplasmic components for cell survival under stress conditions, and triaging of toxic proteins for degradation (Shin and Brangwynne, 2017; Banani et al., 2017; Wang and Zhang, 2019).

Protein LLPS is driven by multivalent weak interactions conferred by modular domains or intrinsically disordered regions (IDRs) (Shin and Brangwynne, 2017; Banani et al., 2017). RNA, either alone or compounded with RNA-binding proteins, also coalesces into liquid-like condensates, such as RNA foci formed by repeat-containing RNAs, stress granules (SGs) and germline granules (Jain and Vale, 2017; Wang and Zhang, 2019). Phase separation is modulated by the composition and concentration of key molecules and also by post-translational modifications (PTMs) that regulate protein-protein or protein-RNA interaction strengths, including phosphorylation, methylation, acylation and ubiquitination (Banani et al., 2017; Shin and Brangwynne, 2017). Protein condensates with liquid properties gradually transition into gel-like and eventually into solid aggregates (Banani et al., 2017; Shin and Brangwynne, 2017). The biophysical properties of protein condensates specify their distinct functions under physiological conditions (Wang and Zhang, 2019).

Polycomb group (PcG) proteins mediate repression of genes related to development and cell fate determination, such as HOX genes (Gellon and McGinnis, 1998; Vidal and Starowicz, 2017). Loss of function of PcG proteins causes widespread ectopic expression of HOX genes, resulting in homeotic transformations (Gellon and McGinnis, 1998; Vidal and Starowicz, 2017). PcG proteins form liquid-like condensates on target gene loci in the nucleus, transforming these regions into transcriptionally silenced heterochromatin (Tatavosian et al., 2019). SOP-2 acts as the *C*. *elegans* functional counterpart of the PRC1 complex to regulate HOX gene expression (Zhang et al., 2003). Loss of *sop*-*2* activity leads to ectopic expression of HOX genes and massive homeotic transformations (Zhang et al., 2003). SOP-2 forms distinct nuclear bodies that appear to be essential for its role in HOX gene repression (Zhang et al., 2003; Zhang et al., 2004a; Zhang et al., 2004b).

We investigated the mechanism by which SOP-2 bodies are assembled. SOP-2::GFP forms distinct spherical structures in the nucleus (Fig. 1A). Fluorescence recovery after photobleaching (FRAP) experiments were performed to examine the internal mobility of SOP-2 bodies. The intensity of the GFP signal quickly recovered to about 48% within 20 s after photobleaching (Fig. 1B and 1C), indicating that SOP-2 proteins in the condensates are highly mobile. Next we examined whether SOP-2 undergoes LLPS *in vitro* (Wang et al., 2019). SOP-2 contains an IDR region and a SAM domain (Fig. 1D). We were unable to obtain full-length SOP-2 with high purity. The IDR region of SOP-2 (referred to as SOP-2(IDR) hereafter) underwent LLPS upon addition of 10% PEG-8000 (Figs. 1E, S1A and S1B). SOP-2(IDR) droplets became larger as the protein concentration increased (Fig. S1C and S1D). FRAP assays showed that the molecules inside SOP-2(IDR) droplets were highly mobile (Fig. 1F and 1G). SOP-2(IDR) droplets underwent extensive fusion (Fig. S1E) and also showed a wetting phenotype on glass slides (Fig. S1F). Formation of SOP-2(IDR) droplets was sensitive to high salt (Fig. S1G–J). SOP-2(IDR) droplets underwent transition over time. In FRAP assays, the recovery rate of the fluorescence signal dramatically slowed down as the droplet induction time was extended (Fig. S1K–M). After induction for 20 min, SOP-2(IDR) droplets rarely fused together upon encounter (Fig. S1N). Thus, SOP-2(IDR) droplets transition into gel-like structures over time. In living animals, SOP-2(IDR)::GFP concentrated into large patches in nuclei (Fig. S1O).

**Figure. 1:**
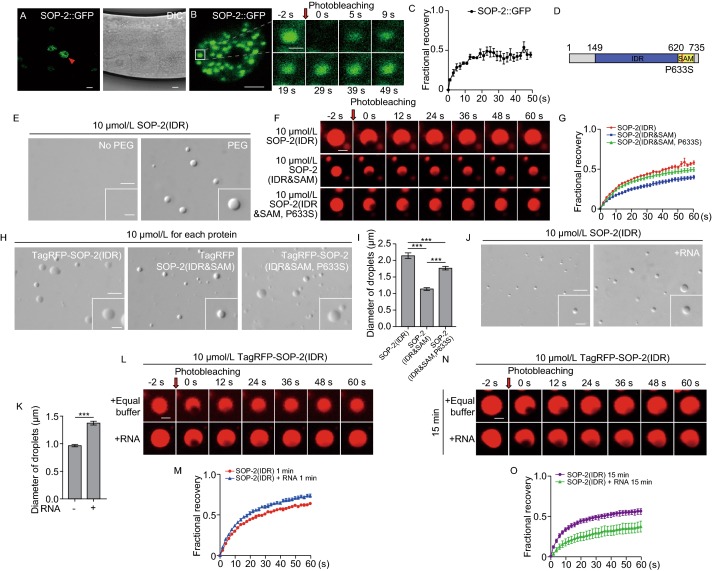
**SOP-2 undergoes liquid-liquid phase separation**. (A) Expression of SOP-2::GFP in *C*. *elegans* hypodermal cells at the young adult stage. SOP-2::GFP localizes in the nucleus and forms spherical structures (marked by red arrow head). The differential interference contrast (DIC) image of the animal is shown on the right. (B and C) FRAP analysis of the SOP-2::GFP signal of the spherical structures in the nucleus of a hypodermal cell in a wild-type animal (B). Quantification of the FRAP data is shown as mean ± SEM (*n* = 3) in (C). The Y axis represents the fractional recovery, which refers to the fraction of the difference between the intensity before and right after the bleaching. (D) Schematic illustration of the SOP-2 protein. The P633S mutation found in *sop*-*2*(*bx91*) disrupts SAM domain-mediated oligomerization of SOP-2. The intrinsically disordered region (IDR) is predicted by PONDR software (http://www.pondr.com/). (E) DIC images showing that 10 μmol/L SOP-2(IDR) undergoes LLPS and forms spherical droplets in buffer containing 10% PEG-8000. (F and G) FRAP analysis of the fluorescence signal of the droplets formed by 10 μmol/L TagRFP-SOP-2(IDR), TagRFP-SOP-2(IDR&SAM) and TagRFP-SOP-2(IDR&SAM, P633S) proteins (F). Quantification of the FRAP data is shown as mean ± SEM (*n* = 6) in (G). (H and I) Compared to droplets formed by 10 μmol/L TagRFP-SOP-2(IDR), the droplets formed by 10 μmol/L TagRFP-SOP-2(IDR&SAM) are smaller, whereas the P633S mutation partially restores the droplet size (H). Droplets formed 1 min after LLPS induction are shown. Quantification of the droplet size in (H) is shown in (I) as mean ± SEM (*n* = 101, 84 and 175 for TagRFP-SOP-2(IDR), TagRFP-SOP-2(IDR&SAM) and TagRFP-SOP-2(IDR&SAM, P633S) droplets, respectively). (J and K) Co-addition of RNA increases the size of the droplets formed by 10 μmol/L SOP-2(IDR) (J). Droplets formed 1 min after LLPS induction are shown. Quantification of the droplet size in (J) is shown in (K) as mean ± SEM (*n* = 148 and 164 for the SOP-2(IDR) and SOP-2(IDR)/RNA droplets, respectively). (L and M) FRAP analysis of the fluorescence signal of the droplets formed by 10 μmol/L TagRFP-SOP-2(IDR) and TagRFP-SOP-2(IDR)/RNA at 1 min after LLPS induction (L). Quantifications of the FRAP data in (L) are shown as mean ± SEM (*n* = 3) in (M). (N and O) FRAP analysis of the fluorescence signal of the droplets formed by 10 μmol/L TagRFP-SOP-2(IDR)/RNA at 15 min after LLPS induction. Quantifications of the FRAP data in (N) are shown as mean ± SEM (*n* = 3) in (O). Scale bars: 5 μm (A, E, H and J), 2 μm (B, F, L and N, inserts in E, H and J) and 0.5 μm (enlarged figures in B)

The SAM domain mediates oligomerization that is important for the function and formation of SOP-2 bodies *in vivo* (Qiao and Bowie, 2005; Zhang et al., 2003). SOP-2(IDR&SAM), containing both IDR and SAM domains of SOP-2, also underwent LLPS *in vitro* (Figs. 1H, S2A and S2B). Compared to SOP-2(IDR) droplets, the SOP-2(IDR&SAM) droplets were smaller, and their formation was also more resistant to high salt (Figs. 1H, 1I and S2C). FRAP assays showed that the internal mobility of SOP-2(IDR&SAM) droplets was significantly decreased compared to SOP-2(IDR) droplets (Figs. 1F, 1G and S2D). The *C*. *elegans* mutant *sop*-*2*(*bx91*) carries a P633S mutation in the SAM domain, which impairs oligomerization of SOP-2 (Zhang et al., 2003) (Fig. 1D). Mutant SOP-2(IDR&SAM, P633S) droplets were larger in size and showed higher mobility in FRAP assays than SOP-2(IDR&SAM) droplets (Figs. 1F–I and S2D). This suggests that SAM domain-mediated oligomerization is important for specifying the biophysical properties of condensates. SOP-2(IDR&SAM)::GFP formed distinct puncta in the nucleus in living animals. When the P633S mutation was introduced, the puncta were still visible, but a diffuse GFP signal was also seen in the nucleus (Fig. S2E and S2F).

SOP-2 binds RNA non-selectively (Zhang et al., 2004a). Deletion of the RNA-binding regions affects the number and size of SOP-2 bodies in *C*. *elegans* (Zhang et al., 2004a). Co-addition of RNA enlarged the size of SOP-2(IDR) and SOP-2(IDR&SAM) droplets (Figs. 1J, 1K, S3A and S3B). FRAP assays showed that co-addition of RNA increased the mobility in SOP-2(IDR) droplets formed immediately after LLPS induction (Figs. 1L, 1M and S3C), while decreasing the mobility in droplets formed 15 min after induction (Figs. 1N, 1O and S3D). Fifteen minutes after induction, SOP-2(IDR) droplets were spherical, while SOP-2(IDR)/RNA droplets became irregularly shaped (Fig. S3E and S3F), indicating slow relaxation of SOP-2(IDR)/RNA droplets after fusion. Thus, RNA promotes gelation of the droplets. Cellular ATP has been shown to act as a biological hydrotrope (Patel et al., 2017). We found that addition of ATP increased the dynamics of the droplets formed by SOP-2(IDR) with or without RNA in the reaction (Figs. S1K–M, S3D, S3G and S3H).

SOP-2 is post-translationally modified by SUMO, which also modulates the formation and functions of SOP-2 bodies (Zhang et al., 2004b). We performed *in vitro* sumoylation assays to determine the residues in SOP-2 that are modified (Flotho et al., 2012). SOP-2 contains three putative sumoylation sites, K73, K453 and K594 (Fig. 2A). Wild-type SOP-2 and SOP-2(IDR) were sumoylated in *in vitro* modification assays (Fig. 2B and S4A). Single mutation of K453R and K594R, but not K73R, impaired sumoylation of SOP-2 (Figs. 2B and S4A). The K453R K594R double mutation further reduced the level of sumoylated SOP-2 (Figs. 2B and S4A), indicating that K453 and K594 of SOP-2 are sumoylated. To examine the effect of sumoylation on LLPS of SOP-2, sumoylated SOP-2(IDR&SAM) and SOP-2(IDR) proteins were subjected to LLPS induction. Droplets formed by sumoylated SOP-2(IDR&SAM) and SOP-2(IDR) were larger in size and more abundant than droplets formed by unmodified proteins (Figs. 2C–E and S4B–D). Sedimentation assays revealed that sumoylated SOP-2(IDR&SAM) and SOP-2(IDR) proteins partitioned into phase-separated droplets (Figs. 2F, S4E and S4F). FRAP assays showed that the internal mobility of SOP-2(IDR&SAM) and SOP-2(IDR) droplets was slightly increased after sumoylation (Figs. 2G, 2H, S4G–J). Thus, sumoylation promotes LLPS of SOP-2. Compared with sumoylated SOP-2(IDR), droplets formed by sumoylated SOP-2(IDR K453R) and SOP-2(IDR K594R) were slightly smaller in size and lower in mobility. The phenotype become more severe when both sites were mutated (Figs. S4B–D and S4G–I). Consistent with the presence of other yet-to-be-identified sumoylation sites in the IDR of SOP-2, droplets formed by sumoylated SOP-2(IDR K453R K594R) were still larger in size and more dynamic compared to the non-modified protein (Fig. S4B–D and S4G–I). In living animals, compared to wild-type SOP-2::GFP, SOP-2(K453R K594R)::GFP bodies were smaller and also displayed a more diffuse GFP signal (Fig. 2I–K).

**Figure. 2:**
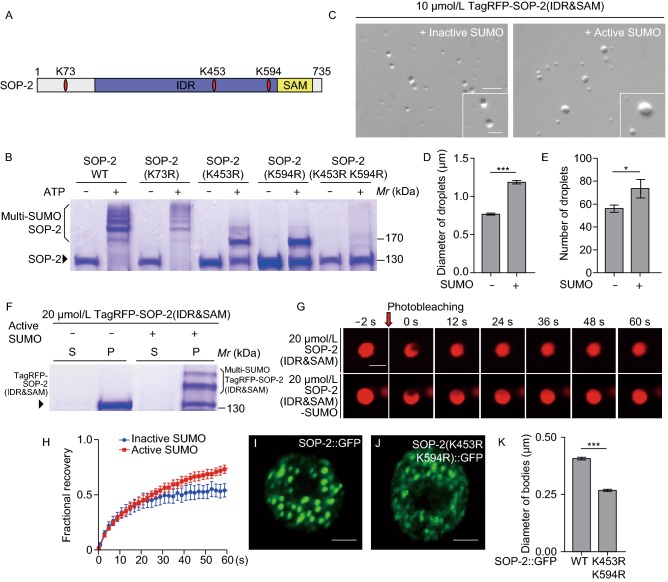
**Sumoylation promotes LLPS of SOP-2**. (A) Schematic illustrating the putative sumoylation sites in SOP-2 that are predicted by GPS-SUMO2.0 software (http://sumosp.biocuckoo.org/online.php). (B) Sumoylation of SOP-2 in the *in vitro* modification assay. The K453R and K594R mutations, but not K73R, impair sumoylation of SOP-2, while the K453R K594R double mutation greatly reduces sumoylation of SOP-2. (C–E) DIC images showing that sumoylation promotes LLPS of TagRFP-SOP-2(IDR&SAM) at 10 μmol/L (C). (D) shows quantification of the size of the unmodified (*n* = 230) and sumoylated (*n* = 232) TagRFP-SOP-2(IDR&SAM) droplets in (C). (E) shows quantification of the number of unmodified and sumoylated droplets in (C), counted from the full area of *n* = 9 and *n* = 7 images, respectively. Proteins were sumoylated and then used for LLPS. Droplets formed 1 min after LLPS are shown. (F) Sedimentation assays showing that sumoylated TagRFP-SOP-2(IDR&SAM) partitions into droplets. (G and H) FRAP analysis of the fluorescence signal of the droplets formed by 20 μmol/L unmodified and sumoylated SOP-2(IDR&SAM) (G). Sumoylation increases the mobility of SOP-2 in the droplets. Quantification of the FRAP data is shown as mean ± SEM (*n* = 3) in (H). (I–K) Expression of SOP-2::GFP (I) and SOP-2(K453R K594R)::GFP (J) in *C*. *elegans* hypodermal cells at the young adult stage. Only the nucleus is shown in the images. Both SOP-2::GFP and SOP-2(K453R K594R)::GFP form spherical structures in the nucleus. The sizes of puncta (I and J) are quantified in (K). Data are shown as mean ± SEM (*n* = 215 and 180 for SOP-2::GFP and SOP-2(K453R K594R)::GFP, respectively). Scale bars: 5 μm (C) and 2 μm (G, I, J and insert in C)

In conclusion, we showed here that the *C*. *elegans* PcG protein SOP-2 undergoes LLPS. The IDR region mediates LLPS of SOP-2, which is modulated by post-translational sumoylation. Oligomerization mediated by the SAM domain accelerates gelation of SOP-2 condensates. RNAs promote LLPS of RNA-binding proteins (Shin and Brangwynne, 2017; Banani et al., 2017). Concentration of specific target mRNAs has also been shown to impact the viscosity and dynamics of droplets formed by the RNA-binding protein Whi3 with a polyQ expansion (Zhang et al., 2015). Our results show that RNAs initially facilitate LLPS and then promote gelation of SOP-2(IDR) droplets. The physiological relevance of the dual function of RNAs in the formation of SOP-2 bodies remains unknown. Repression of HOX genes by SOP-2 may involve multiple steps. RNAs first promote formation of SOP-2 bodies and then reduce their dynamic properties; these changes may be related to the ability of SOP-2 bodies to repress gene expression. Other factors such as ATP and chaperon-like proteins may also modulate the formation and properties of SOP-2 bodies *in vivo*. Our study indicates that oligomerization, post-translational modification and RNA binding modulate LLPS of IDR-containing proteins and also specify the biophysical properties of protein condensates.

## Electronic supplementary material

Below is the link to the electronic supplementary material.

Electronic supplementary material 1 (PDF 1420 kb)

## References

[CR1] Banani SF, Lee HO, Hyman AA, Rosen MK (2017). Biomolecular condensates: organizers of cellular biochemistry. Nat Rev Mol Cell Biol.

[CR2] Flotho A, Werner A, Winter T, Frank AS, Ehret H, Melchior F (2012). Recombinant reconstitution of sumoylation reactions in vitro. Methods Mol Biol.

[CR3] Gellon G, McGinnis W (1998). Shaping animal body plans in development and evolution by modulation of Hox expression patterns. BioEssays.

[CR4] Jain A, Vale RD (2017). RNA phase transitions in repeat expansion disorders. Nature.

[CR5] Patel A, Malinovska L, Saha S, Wang J, Alberti S, Krishnan Y, Hyman AA (2017). ATP as a biological hydrotrope. Science.

[CR6] Qiao F, Bowie JU (2005). The many faces of SAM. Science’s STKE.

[CR7] Shin Y, Brangwynne CP (2017). Liquid phase condensation in cell physiology and disease. Science.

[CR8] Tatavosian R, Kent S, Brown K, Yao T, Duc HN, Huynh TN, Zhen CY, Ma B, Wang H, Ren X (2019). Nuclear condensates of the Polycomb protein chromobox 2 (CBX2) assemble through phase separation. J Biol Chem.

[CR9] Vidal M, Starowicz K (2017). Polycomb complexes PRC1 and their function in hematopoiesis. Exp Hematol.

[CR10] Wang Z, Zhang H (2019). Phase separation, transition, and autophagic degradation of proteins in development and pathogenesis. Trends Cell Biol.

[CR11] Wang Z, Zhang GM, Zhang H (2019). Protocol for analyzing protein liquid-liquid phase separation. Biophys Rep.

[CR12] Zhang H, Azevedo RB, Lints R, Doyle C, Teng Y, Haber D, Emmons SW (2003). Global regulation of Hox gene expression in *C. elegans* by a SAM domain protein. Dev Cell.

[CR13] Zhang H, Christoforou A, Aravind L, Emmons SW, van den Heuvel S, Haber DA (2004). The *C. elegans* Polycomb gene SOP-2 encodes an RNA binding protein. Mol Cell.

[CR14] Zhang H, Elbaum-Garfinkle S, Langdon EM, Taylor N, Occhipinti P, Bridges AA, Brangwynne CP, Gladfelter AS (2015). RNA controls PolyQ protein phase transitions. Mol Cell.

[CR15] Zhang H, Smolen GA, Palmer R, Christoforou A, van den Heuvel S, Haber DA (2004). SUMO modification is required for in vivo Hox gene regulation by the *Caenorhabditis elegans* Polycomb group protein SOP-2. Nat Genet.

