# Supplementary material for: Phase separation of the C. elegans Polycomb protein SOP-2 is modulated by RNA and sumoylation
Source: Protein Cell. 2020 Jan 1;11(3):202–7. doi: 10.1007/s13238-019-00680-y (PMC7026206; doi:10.1007/s13238-019-00680-y)
Supplement: Supplementary file 1 — Electronic supplementary material 1 (PDF 1420 kb) [file 13238_2019_680_MOESM1_ESM.pdf]

Materials and Methods

C. elegans strains

Worms were cultured on King agar medium (2 g/L NaCl, 2.7 g/L peptone, 20 g/L agar, 0.27 g/L Tris-HCl, 0.55 g/L Tris base and 16 g/L cholesterol) plates at 22 ^{o}C and fed with OP50 bacteria. N2 Bristol (*wild type*) and *unc-76(e911)* were used in this study.

Plasmid construction

P*nfya-1*::SOP-2::GFP, P*nfya-1*::SOP-2(IDR)::GFP and P*nfya-1*::SOP-2(IDR&SAM)::GFP were generated by inserting PCR-amplified fragments into the KpnI/SacI sites of Ppd49.26. The plasmid contains 2 kb of the *nfya-1* promoter region. Point mutations were introduced by site-directed mutagenesis.

To construct plasmids expressed in bacteria, PCR-amplified fragments from a *C. elegans* cDNA library were inserted into pET-28a, pACYCDuet-1 and an in-house-modified pET-32a vector, pET-TagRFP-3C (resulting in N-terminal TagRFP-His_6-tagged recombinant proteins). Point mutations were introduced by the method of site-directed mutagenesis.

Generation of transgenic lines

C. elegans transgenic lines were generated by microinjecting the plasmid into *unc-76(e911)* mutant animals. A plasmid expressing *unc-76* was co-injected as the selection marker. *bpEx358* (P*nfya-1*::SOP-2(IDR)::GFP, *unc-76*), *bpEx359* (P*nfya-1*::SOP-2(K453 K594R)::GFP, *unc-76*), *bpEx360* (P*nfya-1*::SOP-2::GFP,

unc-76), *bpEx391* (P*nfya-1*::SOP-2(IDR&SAM)::GFP, *unc-76*) and *bpEx392* (P*nfya-1*::SOP-2(IDR&SAM, P633S)::GFP, *unc-76*) transgenic lines were used in this study.

Protein expression and purification

Recombinant proteins were expressed in *E. coli* CodonPlus (DE3) or TrxB (DE3) strains. Bacterial cells cultured to OD_{600}=0.5-0.6 at 37 ^{o}C were induced by adding 0.3M IPTG, then culture was continued at 18 ^{o}C for 16 h. Cells were collected by centrifugation at 5000 rpm for 15 min, resuspended with binding buffer (50 mM Tris-HCl pH 7.9, 500 mM NaCl and 10 mM imidazole), lysed on a high-pressure homogenizer and centrifuged at 18,000 rpm for 30 min at 4 ^{o}C to pellet the debris. Target proteins in supernatants were affinity purified with Ni-NTA agarose beads (30210, Qiagen). After 3 extensive washes with binding buffer, the bound proteins were eluted with His_6 elution buffer (50 mM Tris-Cl pH 7.9, 500 mM NaCl and 500 mM imidazole). The eluted proteins were loaded onto HiPrep 26/60 Sephacryl S-200 HR columns (17-1195-01, GE Healthcare) on an NGC purifier (BioRAD), and eluted with a buffer containing 25 mM HEPES pH 7.5 and 500mM NaCl. The purified proteins were concentrated to suitable concentrations and finally stored in aliquots at -80 ^{o}C.

***In vitro* phase separation assays**

In vitro phase separation experiments were performed as described (Wang et al., 2019). In brief, to induce LLPS, proteins in buffer containing 25 mM HEPES pH 7.5 and 150 mM NaCl were mixed 1:1 with buffer containing 25 mM HEPES pH 7.5, 150

mM NaCl and 20% PEG-8000 at room temperature. The LLPS systems were pipetted onto a glass slide and DIC or fluorescence images were collected at the indicated time using an M2 microscope (Zeiss). Purified RNAs from HeLa cells were used to examine the effect of RNA on phase separation.

For sedimentation experiments, LLPS was induced for the indicated time at room temperature, and the systems were centrifuged at 13,000 rpm for 5 min. The supernatants and pellets were separated into two tubes and adjusted to an equal volume with SDS loading buffer. After boiling for 5 min, the samples were subjected to SDS-PAGE and Coomassie Brilliant Blue (CBB) staining.

To examine the effect of high salt on LLPS, the induced phase-separated droplets were diluted 2:1 by High Salt buffer (10%PEG-8000 and 3M NaCl).

Time-lapse imaging

Time-series images were taken on a M2 microscope (Zeiss) or LSM880 confocal microscope (Zeiss). Pictures were taken every 2 s during the examination period and processed with ZEN imaging processing software (Carl Zeiss).

For wetting assays, 20 μM TagRFP-SOP-2(IDR) droplets induced for the indicated times were placed on glass-bottomed confocal dishes and filmed on a LSM880 confocal microscope (Zeiss) every 6 s.

Fluorescence recovery after photobleaching (FRAP) assays

The FRAP experiments were performed on a LSM880 confocal microscope (Zeiss). Selected regions were photobleached with a 488 nm laser beam for GFP-tagged proteins or a 588 nm laser beam for TagRFP-tagged proteins. The

fluorescence intensities of selected regions were collected every 2 s as mean ROI. The recovery rate value was normalized to the initial intensity before photobleaching. Curves of recovery rates were generated with GraphPad Prism 5 software.

***In vitro* sumoylation assays**

Bacterially expressed and purified proteins were purified and used for the *in vitro* sumoylation experiments (Flotho et al., 2012). The reactions were performed in sumoylation buffer containing 20 mM HEPES, pH 7.3, 2 mM magnesium acetate, 110 mM potassium acetate, 1 mM DTT, 1 mM EGTA, 0.05% Tween20 and 1 μg/mL each of leupeptin, pepstatin and aprotinin. 5 μg indicated substrate proteins, 200 ng His-Aos1/Uba2 (E1 ligase), 500 ng His-Ubc9 (E2 ligase) and 1 μg active or inactive SUMO1 were mixed in each 20 μl reaction system. 0.5 mM ATP (dissolved in 20 mM HEPES, pH 7.4 and 100 mM magnesium acetate) was then added. After incubation at 30 ^{o}C for 2 h, the reactions were terminated by adding SDS loading buffer and subjected to SDS-PAGE and CBB staining.

For LLPS experiments with sumoylated proteins, each 40 μl reaction system contained 40 μM indicated substrate proteins, 1 μg His-Aos1/Uba2, 2 μg His-Ubc9, 5 μg active or inactive SUMO1 and 0.5 mM ATP. After incubation at 30 ^{o}C for 2 h, the reaction systems were centrifuged at 13,000 rpm for 5 min. The pellets were discarded and the supernatants were used for the LLPS experiments.

Quantification and statistical analysis

The diameter and number of liquid droplets were measured with Image J and statistically analyzed with GraphPad Prism 5. All data are shown as mean ± SEM. The

statistical differences were calculated using the unpaired Student’s t test method. n.s., no significant difference, *, $P<0.05$; **, $P<0.01$; ***, $P<0.001$.

Figure legends

Supplementary figure 1. The IDR region of SOP-2 undergoes LLPS and forms liquid-like condensates.

(A) Schematic illustration of the sedimentation assay. Droplets formed by LLPS are precipitated by centrifugation at 13,000 rpm for 5 min. The supernatants and pellets are adjusted to equal volume, mixed with SDS loading buffer, boiled at 100 ^{o}C for 5 min and then subjected to SDS-PAGE.

(B) Levels of TagRFP-SOP-2(IDR) protein in the solution and droplets, assessed by Coomassie Brilliant Blue (CBB) staining of SDS-PAGE. S, supernatant; P, pellet.

(C and D) Fluorescence images showing that 3, 8, 15 and 20 μM TagRFP-SOP-2(IDR) undergoes LLPS by addition of 10% PEG-8000 (C). Droplets formed 1 min after LLPS induction were imaged and analyzed. Quantification of the droplet size in (C) is shown in (D) as mean ± S.E.M. (n=206, 161, 194 and 207 for droplets formed by 3, 8, 15 and 20 μM TagRFP-SOP-2(IDR), respectively).

(E and F) Droplets formed by 20 μM SOP-2(IDR) undergo fusion (E) and wetting (F) *in vitro*. Droplets formed 1 min after LLPS induction were used for imaging. In (E), “0 s” indicates the first contact between the droplets.

(G-I) Preformed droplets by 10 μM TagRFP-SOP-2(IDR) are largely dissolved by

high salt as shown by imaging (G) and sedimentation experiments (I). Quantification of the droplet size in (G) is shown as mean ± S.E.M. (n=105 and 100 for droplets in low salt and high salt buffer, respectively) in (H). HS: high salt buffer.

(J) Schematic diagram illustrating LLPS of SOP-2(IDR) at various concentrations and in buffers containing various concentrations of NaCl.

(K-M) FRAP analysis of the fluorescence signal of the droplets formed by 10 μM TagRFP-SOP-2(IDR) for the indicated times after induction of LLPS. The bottom panel shows FRAP analysis of droplets induced in the reaction containing 0.5 mM ATP. The droplets undergo gelation with time. Quantification of the FRAP data is shown as mean ± S.E.M. (n=5 for each curve) in (L). The FRAP data were analyzed and presented in (M) as maximum recovery rate of the mobile fraction. Data in (M) are shown as mean ± S.E.M. (n=7, 7, 6, 6, and 7 for droplets formed 10 min, 25 min, 1 h ,4 h and 4 h (containing ATP) after induction, respectively).

(N) Droplets formed by 10 μM TagRFP-SOP-2(IDR) 20 min after LLPS induction fail to fuse upon encounter.

(O) In living animals, SOP-2(IDR)::GFP forms large patches (indicated by the red arrow) in the nucleus of hypodermal cells. Only the nucleus is shown in (O).

Scale bars: 5 μm (C, F and G) and 2 μm (E, K, N, O and inserts in C and G).

Supplementary figure 2. The SAM domain accelerates gelation of SOP-2 condensates.

(A and B) DIC images showing that 3, 5, 15 and 20 μM TagRFP-SOP-2(IDR&SAM) undergoes LLPS by addition of 10% PEG-8000 (A). Droplets formed 1 min after LLPS induction are shown. Quantification of the droplet size in (A) is shown in (B) as mean ± S.E.M. (n=190, 252, 228 and 212 for the concentrations of 3, 5, 15 and 20 μM, respectively).

(C) Preformed droplets by 10 μM TagRFP-SOP-2(IDR&SAM) in buffer containing 150 mM NaCl are partially dissolved by adjusting the NaCl concentration to 1000 mM, as shown by the sedimentation assay.

(D) The FRAP data in Fig. 1G were analyzed and presented as maximum recovery rate of the mobile fraction (Fm). Data are shown as mean ± S.E.M. (n= 6 for each bar).

(E and F) In hypodermal cells, SOP-2(IDR&SAM)::GFP forms distinct and bright spherical structures in the nucleus (E). SOP-2(IDR&SAM, P633S)::GFP also forms spherical structures and also shows a diffuse GFP signal (marked by the white arrowhead) (F).

Scale bars: 5 μm (A) and 2 μm (E, F and insert in A)

Supplementary figure 3. RNA accelerates gelation of SOP-2 condensates.

(A and B) Co-addition of RNA slightly increases the size of the droplets formed by 10 μM TagRFP-SOP-2(IDR&SAM) at 1 min after LLPS induction (A). Quantification of the droplet size in (A) is shown in (B) as mean ± S.E.M. (n=234 and 213 for the TagRFP-SOP-2(IDR&SAM) and

TagRFP-SOP-2(IDR&SAM)/RNA droplets, respectively). Droplets formed 1 min after LLPS induction are shown.

(C) FRAP data of Fig. 1M was analyzed in (C), data was shown as mean ± S.E.M. (n= 3 for each quantification.)

(D) FRAP data of Fig. 1O and Fig. S3H was analyzed in (D), data was shown as mean ± S.E.M. (n= 3, 3, 6 and 8 for no both RNA and ATP, with RNA but no ATP, no RNA but with ATP, with both RNA and ATP respectively.)

(E and F) Compared to droplets formed by 10 μM TagRFP-SOP-2(IDR) 15 min after induction, droplets formed by 10 μM TagRFP-SOP-2(IDR)/RNA are irregularly shaped, which indicates slower relaxation of fused droplets into a spherical structure. Quantification of the droplet size in (E) is shown in (F) as mean ± S.E.M. (n=140 and 161 for the TagRFP-SOP-2(IDR) and TagRFP-SOP-2(IDR)/RNA droplets, respectively).

(G and H) FRAP analysis of the fluorescence signal of the droplets formed by 10 μM TagRFP-SOP-2(IDR) in the reaction with or without 0.5 mM ATP. Droplets formed 15 min after induction were analyzed. Quantification of the FRAP data is shown as mean ± S.E.M. (n=3 for each curve) in (H).

Scale bars: 5 μm (A and E) and 2 μm (G, inserts in A and E).

Supplementary figure 4. Sumoylation modulates LLPS of SOP-2.

(A) Sumoylation of SOP-2(IDR) in *in vitro* modification experiments. The K453R or K594R single mutation impairs sumoylation of SOP-2(IDR), while the K453R

K594R double mutation almost completely abolishes sumoylation of SOP-2(IDR).

(B-D) Fluorescence images showing that sumoylation promotes LLPS of SOP-2(IDR) at 20 μM (D). (B) shows quantification of the size of the droplets formed by unmodified TagRFP-SOP-2(IDR) (n=210), sumoylated TagRFP-SOP-2(IDR) (n=295), sumoylated TagRFP-SOP-2(IDR K453R) (n=198), sumoylated TagRFP-SOP-2(IDR K594R) (n=227), and sumoylated TagRFP-SOP-2(IDR K453R K594R) (n=181) in (D). (C) shows quantification of the number of unmodified and sumoylated droplets in (D) (counted from the full area of n=7, 8, 8, 8 and 9 images for unmodified TagRFP-SOP-2(IDR), sumoylated TagRFP-SOP-2(IDR), sumoylated TagRFP-SOP-2(IDR K453R), sumoylated TagRFP-SOP-2(IDR K594R) and sumoylated TagRFP-SOP-2(IDR K453R K594R), respectively).

(E) Schematic illustration of the sedimentation assay for sumoylated SOP-2 proteins. After sumoylation for 2 h, the system is subjected to centrifugation at 13,000 rpm for 10 min. The subsequent operations are the same as shown in Fig. S1A.

(F) Levels of sumoylated TagRFP-SOP-2(IDR) protein in solution and pellets, detected by sedimentation assays.

(G-I) FRAP analysis of the fluorescence signal of the droplets formed by 10 μM unmodified TagRFP-SOP-2(IDR), sumoylated TagRFP-SOP-2(IDR), sumoylated TagRFP-SOP-2(IDR K453R), sumoylated TagRFP-SOP-2(IDR K594R) and sumoylated TagRFP-SOP-2(IDR K453R K594R) (H). The FRAP data in (G)

were analyzed and presented as maximum recovery rate of the mobile fraction (Fm). Data are shown as mean ± S.E.M. (n= 4 for each sample)

(J) The FRAP data in Fig. 2H were analyzed and presented as maximum recovery rate of the mobile fraction (Fm). Data are shown as mean ± S.E.M. (n= 6 and 5 for non-modified and sumoylated TagRFP-SOP-2(IDR&SAM), respectively).

Scale bars: 10 μm (D), 5 μm (insert in D) and 2 μm (H).

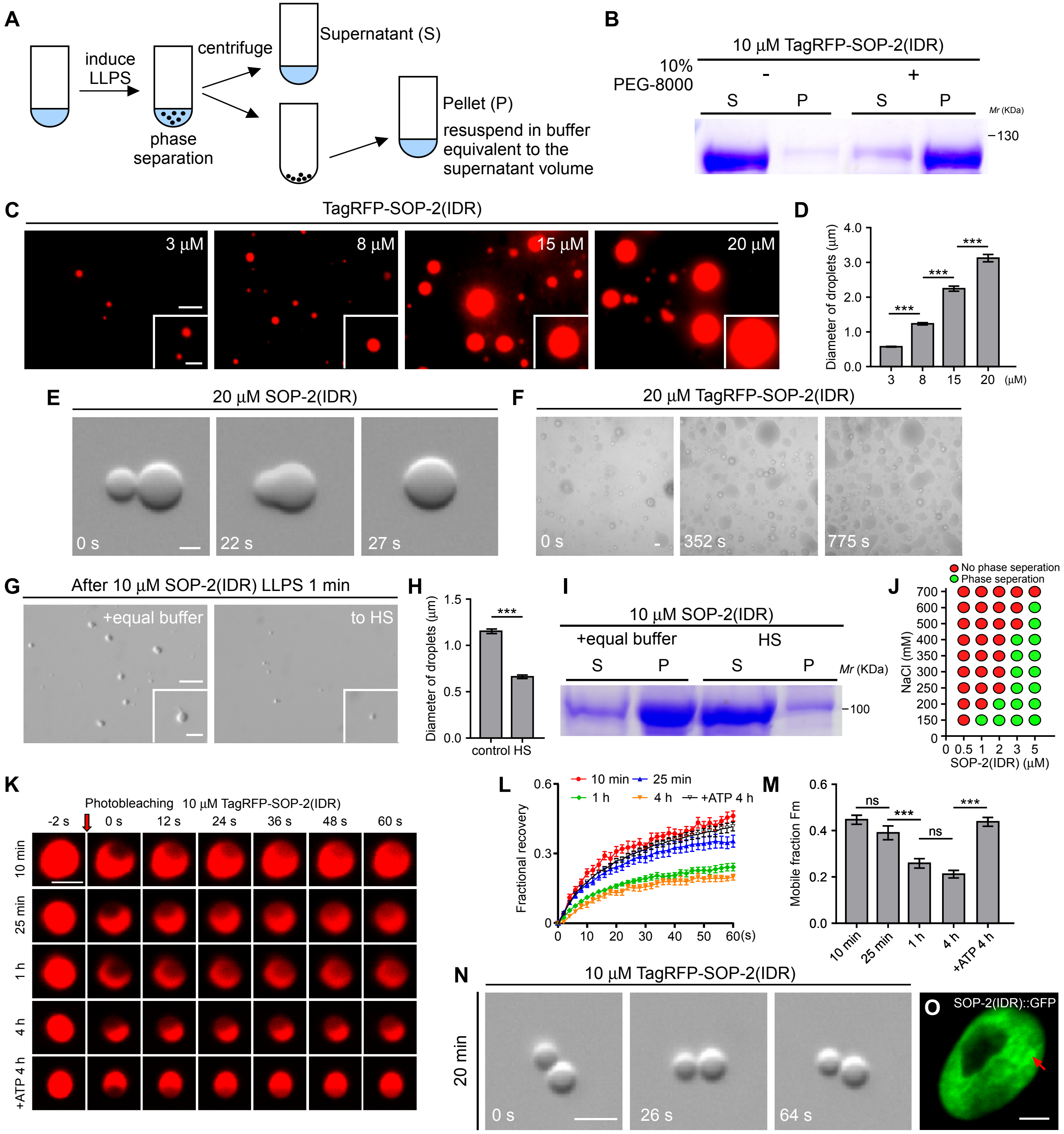
A
induce LLPS
phase separation
centrifuge
Supernatant (S)
Pellet (P)
resuspend in buffer equivalent to the supernatant volume
B
10 µM TagRFP-SOP-2(IDR)
10% PEG-8000
-
+
S P S P
Mr (KDa)
130
C
TagRFP-SOP-2(IDR)
3 µM
8 µM
15 µM
20 µM
D
Diameter of droplets (µm)
***
(µM)
E
20 µM SOP-2(IDR)
0 s
22 s
27 s
F
20 µM TagRFP-SOP-2(IDR)
0 s
352 s
775 s
G
After 10 µM SOP-2(IDR) LLPS 1 min
+equal buffer
to HS
H
Diameter of droplets (µm)
control HS
I
10 µM SOP-2(IDR)
+equal buffer
HS
S P S P
Mr (KDa)
100
J
No phase seperation
Phase seperation
NaCl (mM)
SOP-2(IDR) (µM)
K
Photobleaching 10 µM TagRFP-SOP-2(IDR)
-2 s 0 s 12 s 24 s 36 s 48 s 60 s
10 min
25 min
1 h
4 h
+ATP 4 h
L
Fractional recovery
10 min
25 min
1 h
4 h
+ATP 4 h
60(s)
M
Mobile fraction Fm
ns
***
N
10 µM TagRFP-SOP-2(IDR)
20 min
0 s
26 s
64 s
O
SOP-2(IDR)::GFP

Figure S1

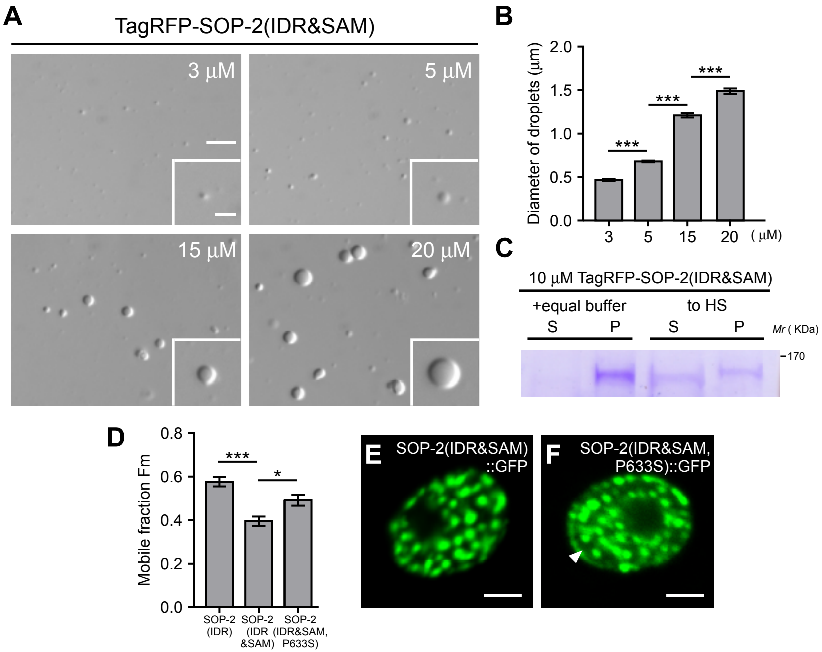
A
TagRFP-SOP-2(IDR&SAM)
3 μM
5 μM
15 μM
20 μM
B
Diameter of droplets (μm)
2.0
1.5
1.0
0.5
0.0
***
***
***
3
5
15
20
(μM)
C
10 μM TagRFP-SOP-2(IDR&SAM)
+equal buffer
to HS
S
P
S
P
Mr (KDa)
170
D
Mobile fraction Fm
0.8
0.6
0.4
0.2
0.0
***
*
SOP-2 (IDR)
SOP-2 (IDR &SAM)
SOP-2 (IDR&SAM, P633S)
E
SOP-2(IDR&SAM) ::GFP
F
SOP-2(IDR&SAM, P633S)::GFP

Figure S2

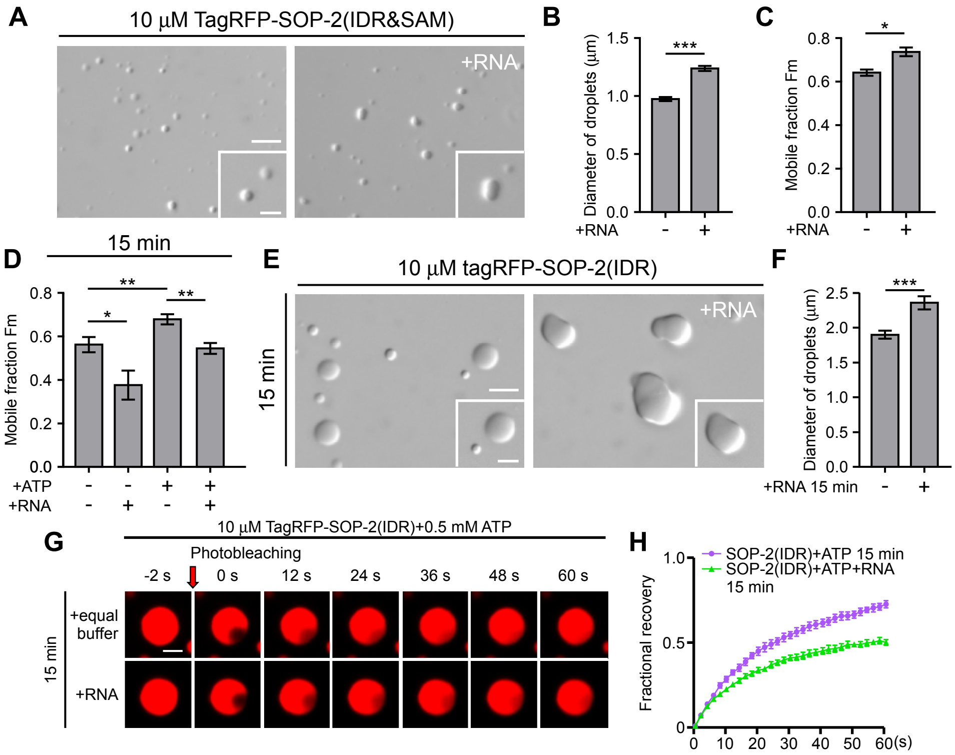
A
10 μM TagRFP-SOP-2(IDR&SAM)
+RNA
B
Diameter of droplets (μm)
1.5
1.0
0.5
0.0
***
+RNA - +
C
Mobile fraction Fm
0.8
0.6
0.4
0.2
0.0
*
+RNA - +
D
15 min
Mobile fraction Fm
**
*
**
+ATP - - + +
+RNA - + - +
E
10 μM tagRFP-SOP-2(IDR)
15 min
+RNA
F
Diameter of droplets (μm)
2.5
2.0
1.5
1.0
0.5
0.0
***
+RNA 15 min - +
G
10 μM TagRFP-SOP-2(IDR)+0.5 mM ATP
Photobleaching
-2 s 0 s 12 s 24 s 36 s 48 s 60 s
15 min
+equal buffer
+RNA
H
SOP-2(IDR)+ATP 15 min
SOP-2(IDR)+ATP+RNA 15 min
Fractional recovery
1.0
0.5
0
0 10 20 30 40 50 60(s)

Figure S3

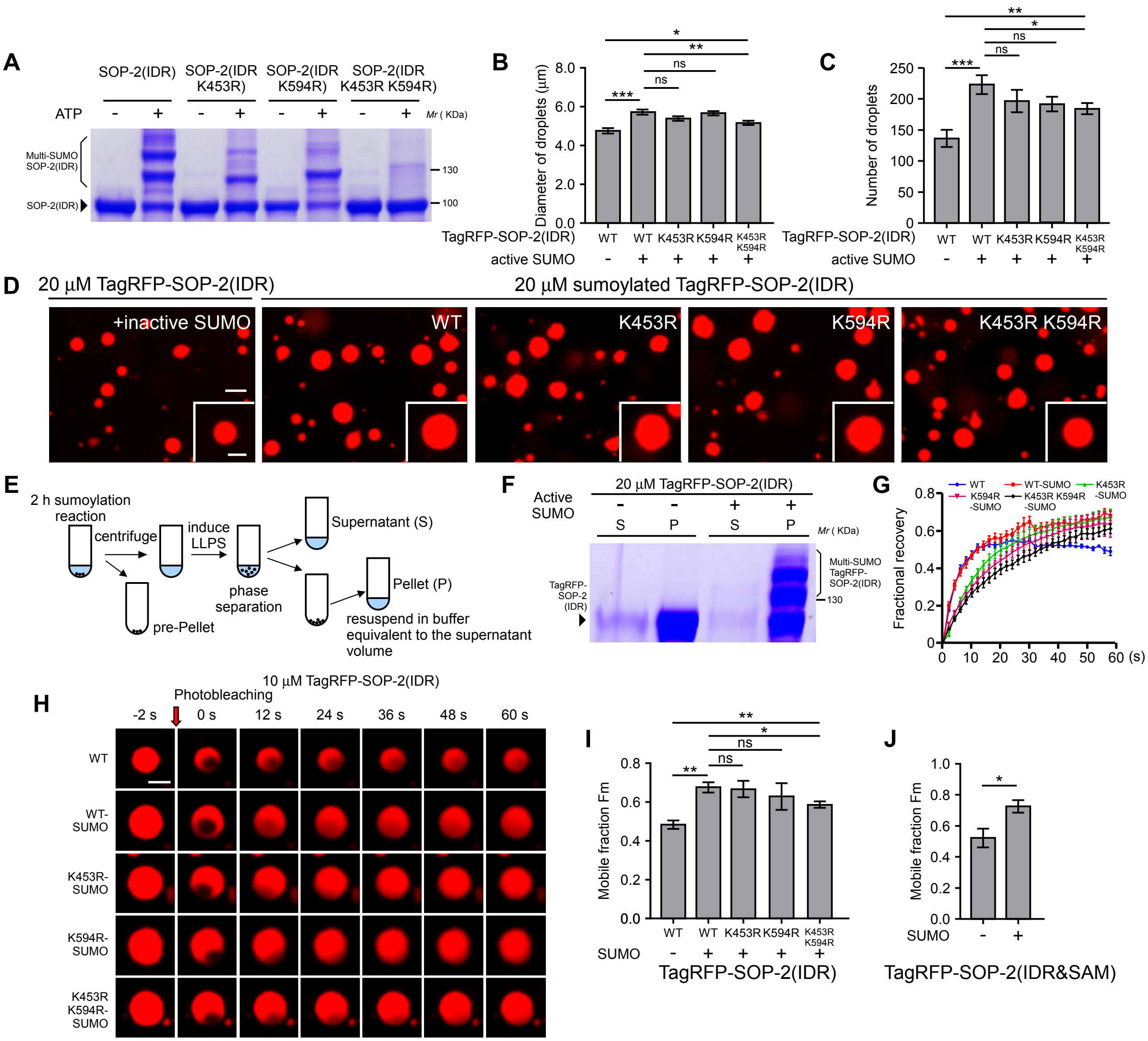
A
SOP-2(IDR)
SOP-2(IDR K453R)
SOP-2(IDR K594R)
SOP-2(IDR K453R K594R)
ATP
Mr (KDa)
Multi-SUMO SOP-2(IDR)
SOP-2(IDR)
130
100
B
Diameter of droplets (μm)
TagRFP-SOP-2(IDR)
active SUMO
WT
K453R
K594R
K453R K594R
C
Number of droplets
D
20 μM TagRFP-SOP-2(IDR)
20 μM sumoylated TagRFP-SOP-2(IDR)
+inactive SUMO
E
2 h sumoylation reaction
centrifuge
induce LLPS
phase separation
Supernatant (S)
Pellet (P)
pre-Pellet
resuspend in buffer equivalent to the supernatant volume
F
Active SUMO
20 μM TagRFP-SOP-2(IDR)
TagRFP-SOP-2 (IDR)
Multi-SUMO TagRFP-SOP-2(IDR)
G
Fractional recovery
WT
WT-SUMO
K453R -SUMO
K594R -SUMO
K453R K594R -SUMO
(s)
H
10 μM TagRFP-SOP-2(IDR)
Photobleaching
-2 s
0 s
12 s
24 s
36 s
48 s
60 s
WT-SUMO
K453R-SUMO
K594R-SUMO
K453R K594R-SUMO
I
Mobile fraction Fm
SUMO
TagRFP-SOP-2(IDR)
J
TagRFP-SOP-2(IDR&SAM)

Figure S4
